# Reassessing the role of antiphospholipid antibodies in placental‐mediated adverse pregnancy outcomes in systemic lupus erythematosus: A retrospective cohort study

**DOI:** 10.1002/ijgo.71147

**Published:** 2026-06-18

**Authors:** Meng Jiang, Yanling Chang, Ning Zhang, Wen Di, Jiayue Wu

**Affiliations:** ^1^ Department of Obstetrics and Gynecology, Renji Hospital, School of Medicine Shanghai Jiao Tong University Shanghai China; ^2^ Shanghai Key Laboratory of Gynecologic Oncology Shanghai China; ^3^ Department of Public Health and Primary Care, Faculty of Medicine and Health Sciences, International Centre for Reproductive Health (ICRH) Ghent University Ghent Belgium; ^4^ State Key Laboratory of Oncogenes and Related Genes, Shanghai Cancer Institute, Renji Hospital, School of Medicine Shanghai Jiao Tong University Shanghai China

**Keywords:** adverse pregnancy outcomes, antiphospholipid antibodies, antiphospholipid syndrome, placental dysfunction, systemic lupus erythematosus

## Abstract

**Objective:**

To examine how different antiphospholipid antibody (aPL) profiles influence placental‐mediated adverse pregnancy outcomes (APOs) in women with systemic lupus erythematosus (SLE).

**Methods:**

This retrospective cohort study included 245 pregnant women with SLE and 490 controls from a single center. Clinical features, immunologic markers and aPL profiles were analyzed. Logistic regression models were used to explore associations between antiphospholipid syndrome (APS), individual aPL subtypes, antibody burden and placental‐mediated APOs.

**Results:**

Placental‐mediated APOs occurred more often in SLE pregnancies than in controls (27.8% vs. 9.8%). After adjustment, APS was not independently associated with APOs. Renal involvement, chronic hypertension and hypocomplementemia remained significant predictors (*P* < 0.05). Among antibody types, lupus anticoagulant (LAC) showed the strongest association in univariate analysis, but the effect weakened in multivariable analysis. Women with multiple aPL positivity had a higher risk of placental complications, whereas single positivity showed little independent effect.

**Conclusion:**

In SLE pregnancies, obstetric risk is not explained by APS alone. Antibody burden, especially multiple aPL positivity, together with renal disease, hypertension and complement levels appears more informative for assessing placental‐related risk.

## 1 INTRODUCTION

Systemic lupus erythematosus (SLE) is a chronic autoimmune disease that primarily affects women of reproductive age. Although multidisciplinary care has improved overall pregnancy outcomes, women with SLE remain at a substantially higher risk of adverse pregnancy outcomes (APOs) compared with the general population. These complications include hypertensive disorders of pregnancy, fetal growth restriction (FGR), preterm birth and fetal loss. Many of these problems are related to placental dysfunction and remain an important cause of maternal and neonatal complications.^1^, ^2^

Antiphospholipid antibody (aPL) is well recognized in the pathogenesis of APOs in women with SLE. The main types include lupus anticoagulant (LAC), anticardiolipin antibody (aCL), and anti‐β2‐glycoprotein 1 antibody (β2GP1). Earlier studies showed that aPLs can impair trophoblast function, activate complement, and promote placental inflammation, leading to placental insufficiency.^3^, ^4^

Antiphospholipid syndrome (APS) is defined by thrombosis and/or obstetric complications together with persistent aPL positivity, such as pre‐eclampsia (PE) and pregnancy loss.^5^, ^6^, ^7^ While APS is known to increase the risk of these outcomes, the role of different aPL patterns in SLE pregnancies remains insufficiently characterized. Furthermore, other factors may affect SLE pregnancy outcomes, including disease activity, low complement levels and antidouble‐stranded DNA (dsDNA) antibodies.^8^, ^9^ Clinical manifestations, such as anemia, thrombocytopenia and renal involvement also contribute to pregnancy risk.^10^ However, most studies have evaluated isolated variables or small groups. Large studies that examine different aPL profiles in SLE are still limited.

This study aimed to characterize the heterogeneity of aPL profiles in pregnant women with SLE, and to reassess the independent contribution of APS and aPL positivity within the context of SLE. Using a large single‐center retrospective cohort and multivariable analysis, we aimed to clarify whether distinct aPL profiles remain strong determinants of placental‐mediated APOs after accounting for SLE‐related clinical and immunologic factors, thereby informing more nuanced risk stratification and individualized pregnancy management.

## 2 MATERIALS AND METHODS

### 2.1 Study design and population

This retrospective cohort study was conducted at Renji Hospital, School of Medicine, Shanghai Jiao Tong University, a tertiary referral center in Shanghai, China. Pregnant women with SLE who were managed and delivered at our institution between January 2020 and December 2024 were consecutively included.

SLE was diagnosed according to the SLICC classification criteria.^11^ Women with multiple gestations, major fetal structural, chromosomal anomalies, incomplete medical records or artificial abortion for personal rather than medical reasons were excluded. A control group was selected from women without autoimmune diseases or APS who received routine prenatal care and delivered during the same period at our center.

Institutional review board approval for this research was granted by the Ethics Committee of Renji Hospital, School of Medicine, Shanghai Jiao Tong University (KY2021‐199‐B). Given the retrospective nature of the study, the requirement for informed consent was waived.

### 2.2 Definition

APS was defined according to the revised Sydney classification criteria, requiring at least one clinical and one laboratory criterion with persistent positivity at least 12 weeks apart.^12^ Laboratory assessment of aPLs included LAC, aCL IgG/IgM, and anti‐β2GP1 IgG/IgM. Titers of ≥40 GPL or MPL units were considered positive for aCL antibodies. APS status was determined based on established clinical diagnosis and available longitudinal laboratory records, rather than solely on aPL results obtained during the index pregnancy.

Disease activity during pregnancy was evaluated using available clinical and laboratory parameters. Immunological markers included complement levels (C3 and C4) and anti‐dsDNA antibodies. Erythrocyte sedimentation rate (ESR) was recorded as a baseline descriptive inflammatory marker, but was not considered a specific indicator of SLE disease activity because of its non‐specific nature during pregnancy. Hypocomplementemia was defined as complement levels below the institutional reference range. Laboratory results used for baseline analyses were obtained from the first available prenatal assessment, usually at 11–13^+6^ weeks of gestation. When repeated quantitative antibody titers were not uniformly available, aPLs were analyzed according to clinically recorded positivity, antibody subtype, and antibody burden. Medication exposure during pregnancy, including glucocorticoids, hydroxychloroquine, aspirin, and low‐molecular‐weight heparin (LMWH), was extracted from medical records and summarized according to clinically documented use during pregnancy.

The primary outcome was placental‐mediated APOs, defined as a composite outcome including: (1) PE, defined according to the ACOG Practice Bulletin criteria as new‐onset hypertension after 20 weeks of gestation accompanied by proteinuria or end‐organ dysfunction.^13^ (2) FGR, defined as estimated fetal weight below the 10th percentile for gestational age in combination with abnormal Doppler findings or other evidence of placental insufficiency, consistent with international consensus recommendations. (3) Early preterm birth (<34 weeks) attributable to placental dysfunction such as severe PE or FGR. (4) Unexplained fetal loss after 10 weeks of gestation, in the absence of chromosomal or structural abnormalities. In addition, late preterm birth (34–36^+6^ weeks) and gestational hypertension (GH) were analyzed.

### 2.3 Statistical analysis

Continuous variables are presented as mean ± standard deviation (SD). Differences between two groups were assessed using the independent‐samples *t*‐test, and comparisons among three or more groups were performed using one‐way analysis of variance (ANOVA) or the Kruskal–Wallis test as appropriate. Categorical variables are expressed as frequencies (percentages) and were compared using the χ^2^ test or Fisher exact test when necessary.

Logistic regression analysis was conducted to evaluate associations between variables and binary outcomes. Variables that were statistically significant in univariate analyses were entered into the multivariable model. Results are presented as odds ratio (OR) with 95% confidence intervals (95% CIs). Model discrimination was assessed using receiver operating characteristic (ROC) curve analysis, and the area under the curve (AUC) was calculated. Model calibration was evaluated using the Hosmer–Lemeshow goodness‐of‐fit test.

Spearman correlation analysis was used to assess associations between continuous variables. All statistical tests were two‐tailed, and a *P* value less than 0.05 was considered statistically significant. Statistical analyses were performed using SPSS version 29.0.

## 3 RESULTS

### 3.1 Baseline characteristics and pregnancy outcomes in women with SLE and healthy controls

A total of 245 women with SLE and 490 healthy controls were included in the analysis. There were no significant differences in maternal age or nulliparity between the two groups. The prevalence of chronic hypertension was higher in women with SLE (5.3% vs. 2.0%, *P* < 0.05), whereas pre‐pregnancy diabetes and history of miscarriage did not differ significantly (Table 1).

**TABLE 1:** Baseline characteristics and pregnancy outcomes in women with SLE and controls.

Variable	SLE (*n* = 245)	Controls (*n* = 490)	*P* value

Maternal age, years	30.44 ± 3.723	30.15 ± 4.448	0.23
Nulliparity	112 (45.7%)	229 (46.7%)	0.79
Previous miscarriage (any)	19 (7.8%)	25 (5.1%)	0.08
Chronic hypertension	13 (5.3%)	10 (2.0%)	0.017
Pre‐pregnancy diabetes	1 (0.4%)	3 (0.6%)	0.59
**Pregnancy complication**			
GH	5 (2.0%)	17 (3.5%)	0.28
PE	42 (17.1%)	27 (5.5%)	<0.001
FGR	35 (14.3%)	26 (5.3%)	<0.001
Late preterm birth^a^	47 (20.5%)	45 (9.3%)	<0.001
Early preterm birth^a^	20 (8.7%)	8 (1.7%)	<0.001
Unexplained fetal loss	8 (3.3%)	6 (1.2%)	0.08
Placental‐mediated APOs	68 (27.8%)	48 (9.8%)	<0.001

*Note*: Values are presented as mean ± SD or *n* (%).

Abbreviations: APOs, adverse pregnancy outcomes; FGR, fetal growth restriction; GH, gestational hypertension; PE, pre‐eclampsia; SD, standard deviation; SLE, systemic lupus erythematosus.

^a^ Calculated among live births.

Placental‐mediated APOs were more frequent in women with SLE than in controls (27.8% vs. 9.8%, *P* < 0.01). The rates of PE, FGR and preterm birth were all significantly higher in the SLE group. Although unexplained fetal loss was numerically more common among women with SLE, the difference did not reach statistical significance (3.3% vs. 1.2%, *P* > 0.05). Similarly, the incidence of GH did not differ significantly between the two groups.

### 3.2 Group profiles in SLE

Patients were categorized into three groups according to clinical aPL/APS status: aPL‐negative without APS, aPL‐positive without APS, and established APS. The baseline characteristics are shown in Table 2.

**TABLE 2:** Baseline characteristics among the three SLE subgroups.

Variable	aPL‐negativity (*n* = 134)	aPL‐positivity without APS (*n* = 82)	APS (*n* = 29)	*P* value
**Maternal characteristics**				
Maternal age, years	30.25 ± 3.721	30.66 ± 3.746	30.66 ± 3.754	0.36
Nulliparity	69 (51.5%)^a^	40 (48.8%)^a^	3 (10.3%)^b^	<0.001
**Clinical manifestations**				
Mucocutaneous disorders	45 (33.6%)	26 (31.7%)	5 (17.2%)	0.22
Arthritis	26 (19.4%)	24 (29.3%)	5 (17.2%)	0.18
Serositis	8 (6.0%)	9 (11.0%)	3 (10.3%)	0.32
Renal involvement	36 (26.9%)^a^	29 (35.4%)^a^	18 (62.1%)^b^	0.001
Anemia	11 (8.2%)	8 (9.8%)	1 (3.4%)	0.67
Thrombocytopenia	3 (2.2%)^a^	8 (9.8%)^b^	3 (10.3%)^b^	0.021
Chronic hypertension	5 (3.7%)^a^	3 (3.7%)^a^	5 (17.2%)^b^	0.026
Previous thrombosis	1 (0.7%)^a^	0 (0%)^a^	2 (6.9%)^b^	0.038
**Immunological indicators**				
Hypocomplementemia‐C3	38 (28.4%)	30 (36.6%)	7 (24.1%)	0.32
Hypocomplementemia‐C4	18 (13.4%)	13 (15.9%)	3 (10.3%)	0.80
Anti‐dsDNA positivity	122 (91.0%)	80 (97.6%)	27 (93.1%)	0.13
ESR, mm/H	32.01 ± 16.08	35.90 ± 24.91	38.86 ± 21.45	0.16
LAC	0 (0%)^a^	80 (97.6%)^b^	21 (72.4%)^d^	<0.001
aCL‐IgG	0 (0%)^a^	3 (3.7%)^b^	5 (17.2%)^d^	<0.001
aCL‐IgM	0 (0%)^a^	2 (2.4%)^b^	2 (6.9%)^b^	0.015
β2GP1‐IgG	0 (0%)^a^	4 (4.9%)^b^	4 (13.8%)^b^	<0.001
β2GP1‐IgM	0 (0%)^a^	10 (12.2%)^b^	4 (13.8%)^b^	<0.001
**Medication during pregnancy**				
Glucocorticoids	128 (95.5%)	81 (98.8%)	29 (100%)	0.38
Hydroxychloroquine	123 (91.8%)	74 (90.2%)	24 (82.8%)	0.30
Aspirin	125 (93.3%)^a^	67 (81.7%)^b^	27 (93.1%)^a^	0.031
LMWH	50 (37.3%)^a^	21 (25.6%)^b^	23 (79.3%)^d^	<0.001

*Note*: Values are presented as mean ± SD or *n* (%). Groups with different superscript letters differ significantly (*P* < 0.05).

Abbreviations: aCL, anticardiolipin antibody; anti‐dsDNA, anti–double‐stranded DNA antibody; aPL, antiphospholipid antibody; APS, antiphospholipid syndrome; β2GP1, anti–β2‐glycoprotein I antibody; ESR, erythrocyte sedimentation rate; LAC, lupus anticoagulant; LMWH, low‐molecular‐weight heparin; SD, standard deviation; SLE, systemic lupus erythematosus.

The three groups were generally comparable in maternal age and overall immunological activity. However, women with APS exhibited a higher prevalence of renal involvement, chronic hypertension, and prior thrombosis. Thrombocytopenia occurred more commonly in the aPL‐positivity and APS groups than in women without aPLs.

As expected, aPL distribution differed markedly across groups, with the highest antibody burden observed in the APS subgroup. The levels of complement, anti‐dsDNA positivity and ESR were comparable among the three groups, suggesting no major difference in baseline immunological and inflammatory profiles. Treatment patterns were largely comparable, although LMWH was used more frequently in APS patients. Overall, these findings indicate that the APS group was characterized by a higher clinical and aPL‐related risk profile.

### 3.3 Impact of aPL heterogeneity on pregnancy outcomes

Pregnancy outcomes differed across the three SLE subgroups (Figure 1). The incidence of placental‐mediated APOs increased progressively from aPL‐negative patients to those with APS, with the highest rates observed in the APS group. A similar trend was observed for PE and preterm birth. Detailed numerical results are provided in Table S1. These findings suggest a gradient effect of aPL status on placental‐mediated complications.

**FIGURE 1:**
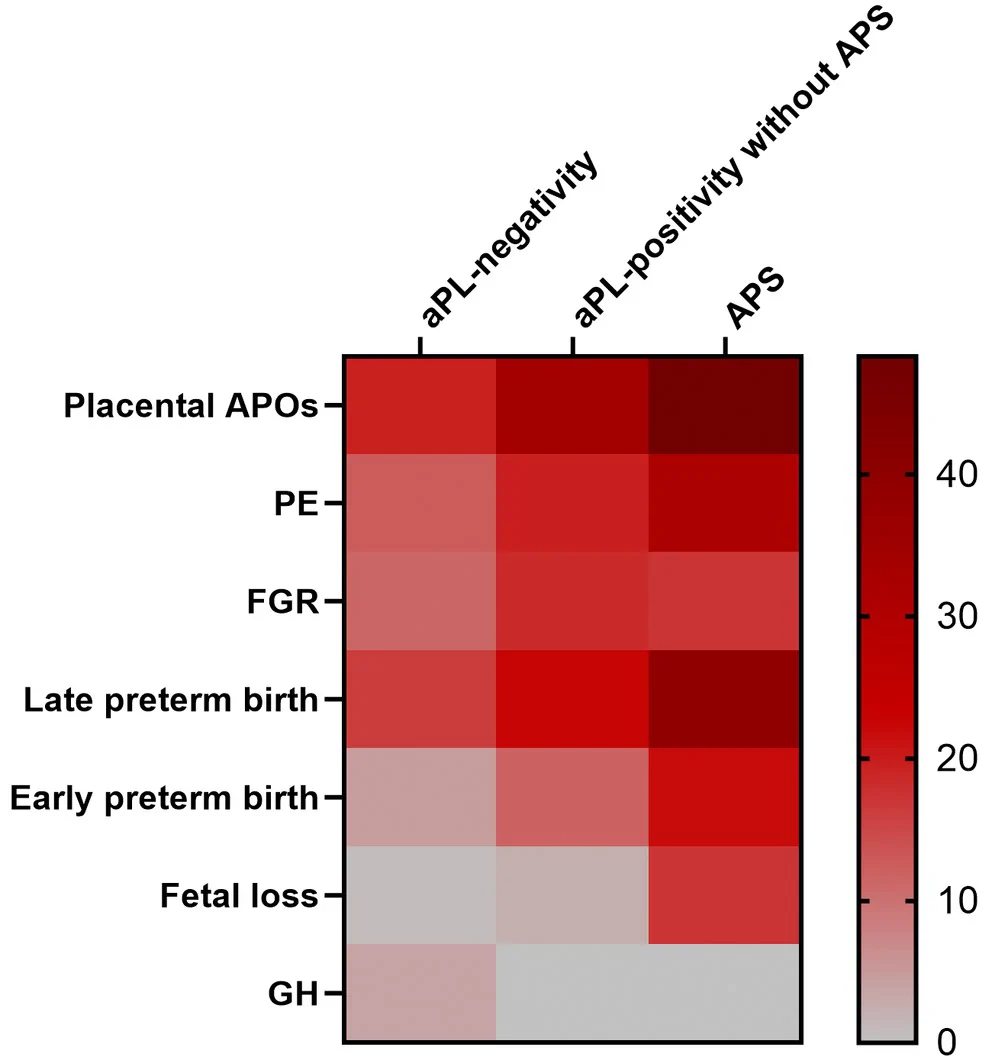
Heatmap of pregnancy outcomes across systemic lupus erythematosus (SLE) subgroups stratified by antiphospholipid antibody (aPL) status. aPL, antiphospholipid antibody; APOs, adverse pregnancy outcomes; APS, antiphospholipid syndrome; FGR, fetal growth restriction; GH, gestational hypertension; PE, preeclampsia.

#### 3.3.1 Association between APS and placental‐mediated APOs

Variables significantly associated with placental‐mediated APOs in univariable analyses (Table S2) were incorporated into the multivariable model. However, after adjustment for clinical factors, APS was no longer an independent predictor (OR 1.48, *P* > 0.05).

In contrast, renal involvement, chronic hypertension, and hypocomplementemia remained significantly associated with APOs (Figure 2). The model demonstrated acceptable discrimination and calibration (Hosmer–Lemeshow *P* = 0.69, AUC = 0.784) (Figure S1). These results suggest that APS alone does not fully explain obstetric risk in SLE.

**FIGURE 2:**
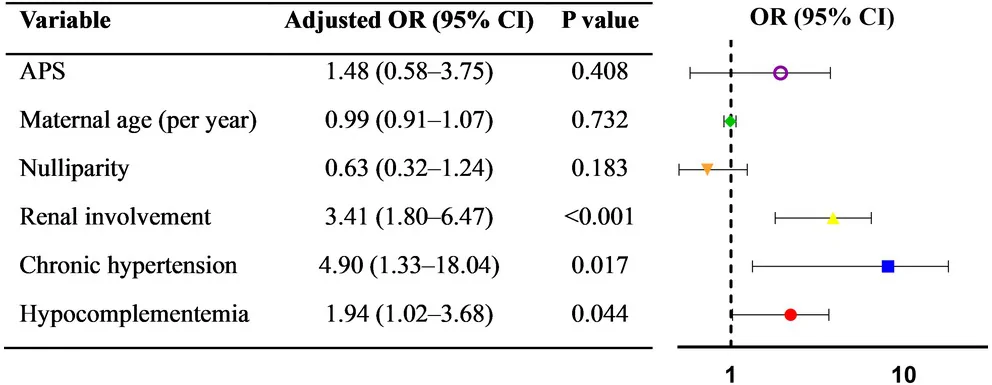
Multivariable logistic regression analysis of factors associated with placental‐mediated adverse pregnancy outcomes (APOs) (Model 1). APOs, adverse pregnancy outcomes; APS, antiphospholipid syndrome; CI, confidence interval; OR, odds ratio. The x‐axis is displayed on a logarithmic scale (base 10). Effect estimates (odds ratios) and their 95% confidence intervals are plotted.

#### 3.3.2 Individual aPL subtypes and placental‐mediated APOs

In univariable analyses, LAC, aCL‐IgG, and β2GP1‐IgG were each significantly associated with placental‐mediated APOs (all *P* < 0.01) (Table S3). To determine which antibody subtype was most strongly associated within the aPL spectrum, all five antibody subtypes were simultaneously entered into a logistic regression model restricted to antibody variables only. Consequently, LAC remained independently associated with placental‐mediated APOs (OR 2.01, *P* < 0.05), whereas the other subtypes lost statistical significance.

However, after adjustment for clinical variables, no individual antibody subtype retained independent significance. LAC showed only a borderline association. Renal involvement, hypertension, and hypocomplementemia remained the dominant predictors (Figure 3). The antibody‐based model demonstrated acceptable discrimination (AUC = 0.759) and good calibration (Hosmer‐Lemeshow *P* = 0.90) (Figure S2). This indicates that the effect of individual aPLs is influenced by the broader clinical context of SLE.

**FIGURE 3:**
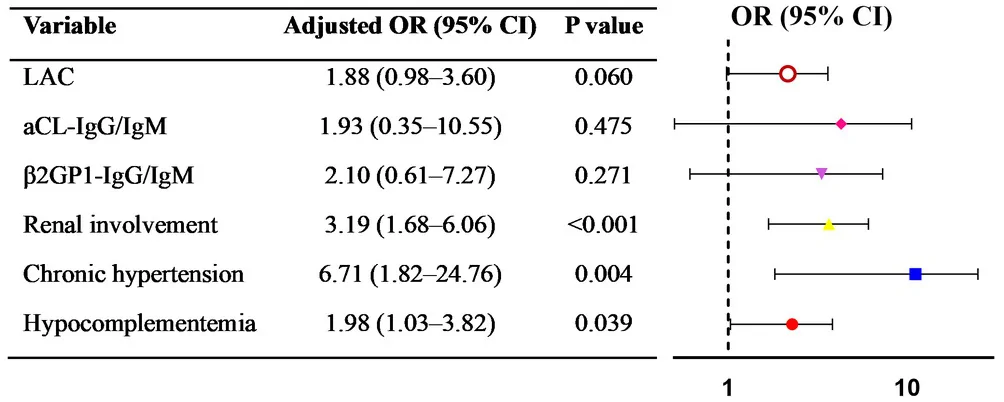
Multivariable logistic regression analysis of individual aPL subtypes and placental‐mediated adverse pregnancy outcomes (APOs) (Model 2). aCL, anticardiolipin antibody; aPL, antiphospholipid antibody; APOs, adverse pregnancy outcomes; CI, confidence interval; LAC, lupus anticoagulant; OR, odds ratio; β2GP1, anti–β2‐glycoprotein I antibody. The x‐axis is displayed on a logarithmic scale (base 10). Effect estimates (odds ratios) and their 95% confidence intervals are plotted.

#### 3.3.3 Association between aPL burden and placental‐mediated APOs

To further explore the impact of antibody heterogeneity, a multivariable logistic regression model was constructed using aPL burden (negative, single positivity, and ≥ double positivity). aPL burden was classified according to aPL results recorded during the index pregnancy, rather than established APS status. The incidence of placental‐mediated APOs increased progressively across aPL‐burden groups (Table S4). Women with multiple aPL positivity had a higher risk of adverse outcomes, whereas single positivity showed no clear independent effect (Figure 4).

**FIGURE 4:**
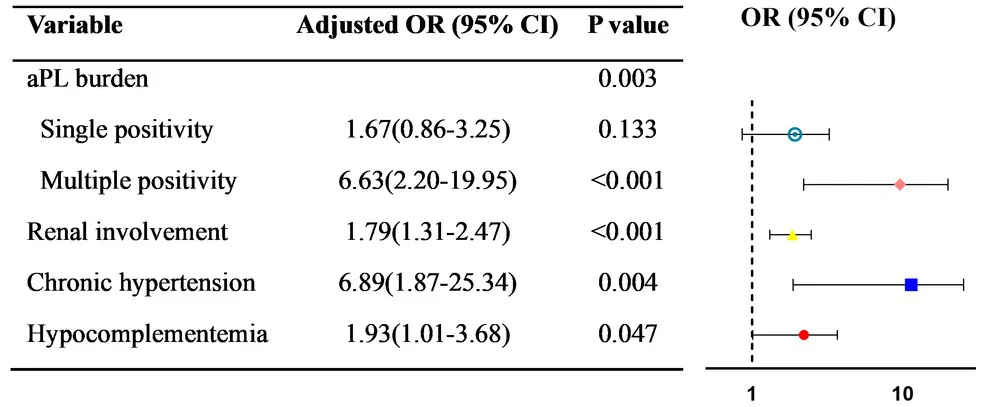
Association between antiphospholipid antibody (aPL) burden and placental‐mediated adverse pregnancy outcomes (APOs) (Model 3). aPL, antiphospholipid antibodies; APOs, adverse pregnancy outcomes; CI, confidence interval; OR, odds ratio. The x‐axis is displayed on a logarithmic scale (base 10). Effect estimates (odds ratios) and their 95% confidence intervals are plotted.

Clinical factors, including renal involvement, chronic hypertension, and hypocomplementemia, remained consistently associated with increased risk. The model showed acceptable discrimination and good calibration (Hosmer‐Lemeshow *P* = 0.96, AUC = 0.757) (Figure S3). These findings highlight the importance of considering overall antibody burden rather than isolated antibody positivity.

## 4 DISCUSSION

The present study examined the role of aPL heterogeneity in placental‐mediated APOs among women with SLE. Our findings suggest that APS alone does not fully account for obstetric risk in this population. Instead, multiple aPL positivity together with key clinical factors such as renal involvement, chronic hypertension and hypocomplementemia, provides a more informative framework for risk assessment.

Previous studies largely focused on patients with aPL/APS or autoimmune populations, in which SLE was only a subgroup.^14^, ^15^, ^16^ In that setting, APS usually appeared as a strong risk factor for poor pregnancy outcomes. However, these designs do not clarify whether APS retains comparable influence in women with SLE, where renal involvement, complement activation, and chronic endothelial injury also contribute to placental dysfunction.

Consistent with previous studies, we confirmed that women with SLE remain at substantially increased risk of PE, FGR, and preterm birth compared with healthy controls.^1^, ^2^, ^17^, ^18^ Although APS has traditionally been considered a major risk factor for APOs, its independent effect was attenuated after adjustment in our cohort. This may reflect the complex pathophysiology of SLE, in which vascular, inflammatory, and immunological factors interact to influence placental function.

Among individual antibodies, LAC showed the strongest association consistent with previous studies.^19^, ^20^ However, its effect weakened in fully adjusted models, suggesting that antibody status alone cannot fully explain placental injury in SLE. Instead, its impact appears to depend on the broader disease context, including complement activation and underlying organ involvement.^21^, ^22^

In contrast, cumulative antibody exposure may better reflect pathogenic potential than individual antibody subtypes. A previous study has already shown that triple aPL positivity has been associated with increased obstetric risk.^23^ Our findings extend this concept to SLE population. Even without fulfilling APS criteria, multiple aPL positivity was independently associated with a stepwise rise in placental‐mediated complications.

These results have practical implications for pregnancy management. Current strategies often focus mainly on APS classification and triple positivity when determining anticoagulation and monitoring intensity. The 2023 EULAR recommendations emphasize preconception disease control and risk‐adapted monitoring in women with SLE, especially in the presence of aPLs or prior nephritis, while acknowledging uncertainty in patients with non‐criteria or isolated antibody positivity.^24^ Our data suggest that in women with SLE, a comprehensive risk assessment might be improved by evaluation of aPL burden alongside disease‐related factors, thereby informing individualized management strategies. Assessing the complete aPL profile may be more effective than relying solely on APS classification in assessing pregnancy risk in the SLE population.

The strengths of the present study include a relatively large SLE cohort, detailed antibody characterization, and hierarchical modeling that distinguishes antibody‐restricted from clinically adjusted effects. Several limitations should be acknowledged. First, as a retrospective single‐center study, treatment decisions were individualized according to disease status, aPL/APS status, and obstetric risk; therefore, the independent effects of specific medications could not be reliably separated from confounding by indication. Second, complete quantitative aPL titers were not available for all patients, and we therefore could not evaluate the relationship between mean antibody levels and placental‐mediated APOs. Instead, we focused on clinically recorded aPL positivity, antibody subtype, and antibody burden, which were more consistently available and relevant to routine clinical risk assessment. Third, detailed neonatal outcomes were not uniformly available. Future prospective studies with standardized medication records, serial quantitative aPL measurements, and detailed neonatal follow‐up are warranted.

## 5 CONCLUSION

In summary, aPL heterogeneity plays a role in placental‐mediated risk in SLE. Multiple aPL positivity appears to be an important factor in risk stratification. Renal involvement, hypertension and hypocomplementemia also confer independent risk. APS alone does not fully define obstetric risk in SLE. A combined assessment of antibody profile with clinical factors and complement levels may provide a more useful framework for individualized pregnancy management.

## AUTHOR CONTRIBUTIONS

Conception and design: M.J, YL.C JY.W. Administrative support: JY.W, W.D. Provision of study materials or patients: M.J, YL.C. Collection and assembly of data: M.J, YL.C, JY.W. Data analysis and interpretation: M.J, YL.C, N.Z, W.D, JY.W. All authors read and approved the final version of the manuscript.

## FUNDING INFORMATION

This work was supported by the National Natural Science Foundation of China (grant nos. 82 271 697 and 82 101 768), the National Key R&D Program of China (grant no. 2024YFC2706700), the Project of College‐Nominated NSFC General Program (grant no. RJTJ25‐MS‐067), and the Project of College‐Nominated NSFC Youth Program (grant no. ygzrtjxm242776).

## CONFLICT OF INTEREST STATEMENT

The authors declare no conflicts of interest.

## Supporting information


**Table S1.** Placental‐mediated outcomes among SLE subgroups stratified by aPL status.
**Table S2.** Univariable analyses of clinical characteristics associated with placental‐mediated adverse pregnancy outcomes in women with SLE.
**Table S3.** Individual aPL subtypes associated with placental‐mediated APOs in women with SLE.
**Table S4.** Baseline aPL burden associated with placental‐mediated APOs in women with SLE.
**Figure S1.** ROC of Model 1.
**Figure S2.** ROC of Model 2.
**Figure S3.** ROC of Model 3.

## Data Availability

Research data are not shared.

## References

[1] Wind M , Fierro JJ , Bloemenkamp KWM , et al. Pregnancy outcome predictors in systemic lupus erythematosus: a systematic review and meta‐analysis. Lancet Rheumatol. 2024;6(10):e667‐e683. 10.1016/S2665-9913(24)00160-7 39153486

[2] Sangah AB , Jabeen S , Hunde MZ , Devi S , Mumtaz H , Shaikh SS . Maternal and fetal outcomes of SLE in pregnancy: a literature review. Journal of Obstetrics and Gynaecology. 2023;43(1):2205513. 10.1080/01443615.2023.2205513 37154805

[3] Fujieda Y , Amengual O , Atsumi T . Pathogenic role of antiphospholipid antibodies: an update. Lupus. 2018;27(13):2012‐2013. 10.1177/0961203318802016 30282558

[4] Erton ZB , Sevim E , de Jesús GR , et al. Pregnancy outcomes in antiphospholipid antibody positive patients: prospective results from the AntiPhospholipid Syndrome Alliance for Clinical Trials and InternatiOnal Networking (APS ACTION) Clinical Database and Repository ('Registry'). Lupus Sci Med. 2022;9(1):e000633. 10.1136/lupus-2021-000633 PMC9198709 35701043

[5] Li X , Shopit A , Wang J . Biochemical and clinical predictors in pregnant women with antiphospholipid syndrome and systemic lupus erythematosus: comprehensive update. Archives of Gynecology and Obstetrics. 2021;304(5):1153‐1160. 10.1007/s00404-021-06178-5 34390384

[6] Murvai VR , Galiș R , Panaitescu A , et al. Antiphospholipid syndrome in pregnancy: a comprehensive literature review. BMC Pregnancy and Childbirth. 2025;25(1):337. 10.1186/s12884-025-07471-w PMC11934569 40128683

[7] Soto‐Peleteiro A , Gonzalez‐Echavarri C , Ruiz‐Irastorza G . Obstetric antiphospholipid syndrome. Medicina Clínica (Barcelona). 2024;163(1):S14‐S21. 10.1016/j.medcli.2024.05.001 39174149

[8] Tan Y , Yang S , Liu Q , et al. Pregnancy‐related complications in systemic lupus erythematosus. Journal of Autoimmunity. 2022;132:102864. 10.1016/j.jaut.2022.102864 35872104

[9] Liu H , Li M , Wang M , et al. Risk factors for adverse maternal and fetal outcomes in SLE patients: a systematic review and meta‐analysis. Front Med (Lausanne). 2025;12:1573573. 10.3389/fmed.2025.1573573 PMC12504262 41070067

[10] Zucchi D , Fischer‐Betz R , Tani C . Pregnancy in systemic lupus erythematosus. Best Practice & Research. Clinical Rheumatology. 2023;37(4):101860. 10.1016/j.berh.2023.101860 37596173

[11] Petri M , Orbai AM , Alarcón GS , et al. Derivation and validation of the Systemic Lupus International Collaborating Clinics classification criteria for systemic lupus erythematosus. Arthritis and Rheumatism. 2012;64(8):2677‐2686. 10.1002/art.34473 PMC3409311 22553077

[12] Miyakis S , Lockshin MD , Atsumi T , et al. International consensus statement on an update of the classification criteria for definite antiphospholipid syndrome (APS). Journal of Thrombosis and Haemostasis. 2006;4(2):295‐306. 10.1111/j.1538-7836.2006.01753.x 16420554

[13] Gestational Hypertension and Preeclampsia: ACOG Practice Bulletin Summary, Number 222. Obstetrics and Gynecology. 2020;135(6):1492‐1495. 10.1097/AOG.0000000000003892 32443077

[14] Moyle KA , Branch DW , Peterson LK , et al. Association Between Novel Antiphospholipid Antibodies and Adverse Pregnancy Outcomes. Obstetrics and Gynecology. 2025;145(1):55‐64. 10.1097/AOG.0000000000005729 PMC11643356 39666985

[15] Qi W , Zhao J , Huang C , et al. Clinical characteristics and prognosis of patients with antiphospholipid antibodies based on cluster analysis: an 8‐year cohort study. Arthritis Research & Therapy. 2022;24(1):140. 10.1186/s13075-022-02814-w PMC9188169 35690831

[16] Parepalli A , Sarode R , Kumar S , Nelakuditi M , Kumar MJ . Antiphospholipid Syndrome and Catastrophic Antiphospholipid Syndrome: A Comprehensive Review of Pathogenesis, Clinical Features, and Management Strategies. Cureus. 2024;16(8):e66555. 10.7759/cureus.66555 PMC11381965 39252716

[17] Petri M . Pregnancy and Systemic Lupus Erythematosus. Best Practice & Research. Clinical Obstetrics & Gynaecology. 2020;64:24‐30. 10.1016/j.bpobgyn.2019.09.002 31677989

[18] Braga A , Barros T , Faria R , et al. Systemic lupus erythematosus and pregnancy: A retrospective single‐center study of 215 pregnancies from Portugal. Lupus. 2021;30(13):2165‐2175. 10.1177/09612033211050340 34693803

[19] Huang J , Zhu Q , Wang B , et al. Antiphospholipid antibodies and the risk of adverse pregnancy outcomes in patients with systemic lupus erythematosus: a systematic review and meta‐analysis. Expert Review of Clinical Immunology. 2024;20(7):793‐801. 10.1080/1744666X.2024.2324005 38445835

[20] Fazzari MJ , Guerra MM , Salmon J , Kim MY . Adverse pregnancy outcomes in women with systemic lupus erythematosus: can we improve predictions with machine learning? Lupus Sci Med. 2022;9(1):e000769. 10.1136/lupus-2022-000769 PMC9476149 36104120

[21] Kim MY , Guerra MM , Kaplowitz E , et al. Complement activation predicts adverse pregnancy outcome in patients with systemic lupus erythematosus and/or antiphospholipid antibodies. Annals of the Rheumatic Diseases. 2018;77(4):549‐555. 10.1136/annrheumdis-2017-212224 PMC6037302 29371202

[22] Chighizola CB , Lonati PA , Trespidi L , Meroni PL , Tedesco F . The Complement System in the Pathophysiology of Pregnancy and in Systemic Autoimmune Rheumatic Diseases During Pregnancy. Frontiers in Immunology. 2020;11:2084. 10.3389/fimmu.2020.02084 PMC7481445 32973817

[23] Iordache O , Anastasiu DM , Kakarla M , et al. Influence of Antiphospholipid Antibody‐Associated Thrombophilia on the Risk of Preterm Birth: A Systematic Review. Journal of Clinical Medicine. 2023;12(16):5316. 10.3390/jcm12165316 PMC10456069 37629357

[24] Fanouriakis A , Kostopoulou M , Andersen J , et al. EULAR recommendations for the management of systemic lupus erythematosus: 2023 update. Annals of the Rheumatic Diseases. 2024;83(1):15‐29. 10.1136/ard-2023-224762 37827694

