# Supplementary material for: Reassessing the role of antiphospholipid antibodies in placental‐mediated adverse pregnancy outcomes in systemic lupus erythematosus: A retrospective cohort study
Source: Int J Gynaecol Obstet. 2026 Jun 18;175(3):1314–20. doi: 10.1002/ijgo.71147 (PMC13769360; doi:10.1002/ijgo.71147)
Supplement: Supplementary file 1 [file IJGO-175-1314-s001.docx]

**Supplementary Table S1. Placental-mediated outcomes among SLE subgroups stratified by aPL status**

| **Outcome** | **aPL-negativity**  **(n=134)** | **aPL-positivity without APS**  **(n=82)** | **APS**  **(n=29)** | **Total**  **N** | **P value** |
| --- | --- | --- | --- | --- | --- |
| **Primary outcome** |  |  |  |  |  |
| Placental-mediated APOs | 26(19.4%)^a^ | 28(34.1%)^b^ | 14(48.3%)^b^ | 68 | 0.002 |
| **Hypertensive disorders** |  |  |  |  |  |
| GH | 5(3.7%) | 0(0%) | 0(0%) | 5 | 0.20 |
| PE | 17(12.7%)^a^ | 16(19.5%) | 9(31.0%)^b^ | 42 | 0.046 |
| **Fetal outcomes** |  |  |  |  |  |
| FGR | 15(11.2%) | 15(18.3%) | 5(17.2%) | 35 | 0.31 |
| late preterm birth^c^ | 21(16.2%)^a^ | 17(22.4%) | 9(39.1%)^b^ | 47 | 0.002 |
| Early preterm birth^c^ | 6(4.6%)^a^ | 9(11.8%) | 5(21.7%)^b^ | 20 | 0.013 |
| Unexplained fetal loss | 1(0.7%)^a^ | 2(2.4%)^a^ | 5(17.2%)^b^ | 8 | <0.001 |

Values are presented as n (%) in each column; Groups with different superscript letters differ significantly (P < 0.05); c: Calculated among live births.

SLE: systemic lupus erythematosus; aPL: antiphospholipid antibody; APS: antiphospholipid syndrome; APOs: adverse pregnancy outcomes; GH: gestational hypertension; PE: preeclampsia; FGR: fetal growth restriction.

**Supplementary Table S2. Univariable analyses of clinical characteristics associated with placental-mediated adverse pregnancy outcomes in women with SLE**

| **Variable** | **Placental-mediated APOs** | | **PE** | | **FGR** | | **Unexplained fetal loss** | | **Early preterm birth^c^** | |
| --- | --- | --- | --- | --- | --- | --- | --- | --- | --- | --- |
|  | n (%) | P value | n (%) | P value | n (%) | P value | n (%) | P value | n (%) | P value |
| Nulliparity | 24(21.4%) | 0.042 | 14(12.5%) | 0.07 | 14(12.5%) | 0.46 | 1(0.9%) | 0.07 | 4(3.7%) | 0.012 |
| Mucocutaneous disorders | 22(28.9%) | 0.78 | 14(18.4%) | 0.72 | 13(17.1%) | 0.39 | 2(2.6%) | 0.71 | 7(9.5%) | 0.78 |
| Arthritis | 18(32.7%) | 0.35 | 11(20%) | 0.52 | 5(9.1%) | 0.21 | 5(9.1%) | 0.016 | 4(8.2%) | 0.57 |
| Serositis | 9(45%) | 0.07 | 6(30%) | 0.12 | 4(20%) | 0.50 | 1(5%) | 0.49 | 4(2.1%) | 0.07 |
| Renal involvement | 39(47%) | <0.001 | 32(38.6%) | <0.001 | 16(19.3%) | 0.11 | 6(7.2%) | 0.02 | 12(16%) | 0.007 |
| Anemia | 6(30%) | 0.82 | 5(25%) | 0.35 | 1(5%) | 0.32 | 0(0%) | 0.50 | 2(11.1%) | 0.66 |
| Thrombocytopenia | 7(50%) | 0.07 | 5(35.7%) | 0.07 | 2(14.3%) | 0.68 | 2(14.3%) | 0.07 | 2(20%) | 0.21 |
| Chronic hypertension | 9(69.2%) | 0.002 | 7(53.8%) | 0.002 | 3(23.1%) | 0.41 | 1(7.7%) | 0.36 | 4(36.7%) | 0.009 |
| Previous thrombosis | 2(66.7%) | 0.18 | 1(33.3%) | 0.43 | 2(66.7%) | 0.05 | 0(0%) | 0.91 | 1(33.3%) | 0.24 |
| APS | 14(48.3%） | 0.009 | 9(31.0%) | 0.06 | 5(17.2%) | 0.58 | 5(17.2%) | <0.001 | 5(21.7%) | 0.036 |
| Hypocomplementemia | 33(40.7%) | 0.001 | 22(27.2%) | 0.003 | 16(19.8%) | 0.08 | 5(6.2%) | 0.12 | 9(12.9%) | 0.14 |
| Anti-dsDNA positivity | 64(27.9%) | 0.53 | 40(17.5%) | 0.46 | 33(14.4%) | 0.59 | 7(3.1%) | 0.42 | 20(9.3%) | 0.37 |
| LAC | 39(38.6%) | 0.001 | 23(22.8%) | 0.06 | 19(18.8%) | 0.09 | 6(5.9%) | 0.07 | 13(14.3%) | 0.016 |
| aCL-IgG/IgM | 8(72%) | <0.001 | 8(72.7%) | <0.001 | 1(9.1%) | 0.51 | 4(36.4%) | <0.001 | 1(14.3%) | 0.47 |
| aCL-IgG | 8(100%) | <0.001 | 8(100%) | <0.001 | 1(12.5%) | 0.68 | 4(50%) | <0.001 | 1(25%) | 0.31 |
| aCL-IgM | 1(25%) | 0.69 | 1(25%) | 0.53 | 0(0%) | 0.54 | 1(25%) | 0.13 | 0(0%) | 0.76 |
| β2GP1-IgG/IgM | 12(60%) | <0.001 | 9(45%) | 0.002 | 2(10%) | 0.74 | 5(25%) | <0.001 | 3(20%) | 0.13 |
| β2GP1-IgG | 7(87.5%) | <0.001 | 7(87.5%) | <0.001 | 0(0%) | 0.61 | 4(50%) | <0.001 | 0(0%) | 0.69 |
| β2GP1-IgM | 7(50%) | 0.07 | 4(28.6%) | 0.27 | 2(14.3%) | 0.68 | 3(21.4%) | 0.007 | 3(27.3%) | 0.06 |
| aPL burden^d^ |  | <0.001 |  | <0.001 |  | 0.25 |  | <0.001 |  | 0.011 |
| aPL-negativity | 29(20.4%) |  | 19(13.4%) |  | 16(11.3%) |  | 2(1.4%) |  | 7(5.1%) |  |
| Single aPL positivity | 26(31%) |  | 13(15.5%) |  | 16(19%) |  | 1(1.2%) |  | 9(11.4%) |  |
| ≥ Double aPL positivity | 13(68.4%) |  | 10(52.6%) |  | 3(15.8%) |  | 5(26.3%) |  | 4(28.6%) |  |

Values are presented as n (%) in each row; c: Calculated among live births. d: P value refers to comparison across the three categories.

APOs: adverse pregnancy outcomes; GH: gestational hypertension; PE: preeclampsia; FGR: fetal growth restriction; APS: antiphospholipid syndrome; LAC: lupus anticoagulant; aCL: anticardiolipin antibody; β2GP1: anti–β2-glycoprotein I antibody; aPL: antiphospholipid antibody.

**Supplementary Table S3. Individual aPL subtypes associated with placental-mediated APOs in women with SLE**

| **Variable** | **Placental-mediated APOs** | | **P value** |
| --- | --- | --- | --- |
|  | **Yes (n, %)** | **No (n,%)** |  |
| LAC | 39(57.4%) | 62(35%) | 0.001 |
| aCL-IgG | 8(11.8%) | 0(0%) | <0.001 |
| aCL-IgM | 1(1.5%) | 3(1.7%) | 0.69 |
| β2GP1-IgG | 7(10.3%) | 1(0.6%) | <0.001 |
| β2GP1-IgM | 7(10.3%) | 7(4%) | 0.07 |

Values are presented as n (%) in each column; aPL: antiphospholipid antibody; APOs: adverse pregnancy outcomes; SLE: systemic lupus erythematosus; LAC: lupus anticoagulant; aCL: anticardiolipin antibody; β2GP1: anti–β2-glycoprotein I antibody.

**Supplementary Table S4. Baseline aPL burden associated with placental-mediated APOs in women with SLE**

| **Variable** | **aPL non-positivity**  **N=142** | **Single aPL positivity**  **N=84** | **Multiple aPLs positivity**  **N=19** |
| --- | --- | --- | --- |
| **Placental-mediated APOs** | 29(20.4%)^a^ | 26(31.0%)^a^ | 13(68.4%)^b^ |
| PE | 19(13.4%)^a^ | 13(15.5%)^a^ | 10(52.6%)^b^ |
| FGR | 16(11.3%) | 16(19.0%) | 3(15.3%) |
| Early preterm birth^c^ | 7(5.1%)^a^ | 9(11.4%) | 4(28.6%)^b^ |
| Unexplained fetal loss | 2(1.4%)^a^ | 1(1.2%)^a^ | 5(26.3%)^b^ |

Baseline aPL burden was classified according to aPL results recorded during the index pregnancy, rather than established APS status.

Values are presented as n (%) in each column; Groups with different superscript letters differ significantly (P < 0.05); c: Calculated among live births.

aPL: antiphospholipid antibody; APOs: adverse pregnancy outcomes; SLE: systemic lupus erythematosus; PE: preeclampsia; FGR: fetal growth restriction.

**Supplementary Figure S1. ROC of Model 1**

**Supplementary Figure S2. ROC of Model 2**

**Supplementary Figure S3. ROC of Model 3**
